# Inflammatory activation of endothelial cells increases glycolysis and oxygen consumption despite inhibiting cell proliferation

**DOI:** 10.1002/2211-5463.13174

**Published:** 2021-05-12

**Authors:** Jonas Aakre Wik, Danh Phung, Shrikant Kolan, Guttorm Haraldsen, Bjørn Steen Skålhegg, Johanna Hol Fosse

**Affiliations:** ^1^ Department of Pathology Oslo University Hospital‐Rikshospitalet Norway; ^2^ Department of Pathology Institute of Clinical Medicine University of Oslo Norway; ^3^ K.G Jebsen Inflammation Research Centre Institute of Clinical Medicine Faculty of Medicine University of Oslo Norway; ^4^ Department of Nutrition Division of Molecular Nutrition Institute of Basic Medical Sciences University of Oslo Norway

**Keywords:** endothelial cells, glycolysis, IL‐1β, inflammation, metabolism

## Abstract

Endothelial cell function and metabolism are closely linked to differential use of energy substrate sources and combustion. While endothelial cell migration is promoted by 2‐phosphofructokinase‐6/fructose‐2,6‐bisphosphatase (PFKFB3)‐driven glycolysis, proliferation also depends on fatty acid oxidation for dNTP synthesis. We show that inflammatory activation of human umbilical vein endothelial cells (HUVECs) by interleukin‐1β (IL‐1β), despite inhibiting proliferation, promotes a shift toward more metabolically active phenotype. This was reflected in increased cellular glucose uptake and consumption, which was preceded by an increase in PFKFB3 mRNA and protein expression. However, despite a modest increase in extracellular acidification rates, the increase in glycolysis did not correlate with extracellular lactate accumulation. Accordingly, IL‐1β stimulation also increased oxygen consumption rate, but without a concomitant rise in fatty acid oxidation. Together, this suggests that the IL‐1β‐stimulated energy shift is driven by shunting of glucose‐derived pyruvate into mitochondria to maintain elevated oxygen consumption in HUVECs. We also revealed a marked donor‐dependent variation in the amplitude of the metabolic response to IL‐1β and postulate that the donor‐specific response should be taken into account when considering targeting dysregulated endothelial cell metabolism.

## Graphical abstract

Inflammatory activation of human umbilical vein endothelial cells (HUVECs) by interleukin‐1β (IL‐1β) increases glucose uptake and consumption, and simultaneously inhibits cell proliferation. Despite reducing fatty acid oxidation, IL‐1β also increases oxygen consumption rates while maintaining extracellular lactate levels. Our observations suggest that increased glycolysis in IL‐1β‐stimulated HUVECs predominantly fuels an increase in mitochondrial pyruvate metabolism.

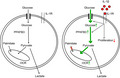

**Abbreviations**
ECAR: extracellular acidification rate
F‐2,6‐BP: fructose‐2,6‐bisphosphate
FAO: fatty acid oxidation
FCCP: carbonyl cyanide‐4‐(trifluoromethoxy)phenylhydrazone
HUVEC: human umbilical vein endothelial cells
IL‐1β: interleukin‐1β
OCR: oxygen consumption rate
PFK‐1: phosphofructokinase 1
PFKFB3: 2‐phosphofructokinase‐6/fructose‐2,6‐bisphosphatase 3
TUNEL: terminal deoxynucleotidyl transferase dUTP nick end labeling
VCAM‐1: vascular cell adhesion molecule 1

While endothelial cells in healthy, noninfected tissues are in proliferative quiescence, they actively maintain anticoagulant blood flow, vascular tone, and a strict regulation of transport across the vessel wall. In response to extracellular events that induce angiogenic or pro‐inflammatory signals, endothelial cells respond to take on new functions [1, 2]. For example, in response to signaling from hypoxic cells, dynamic tip and stalk cell phenotypes arise from capillary endothelium and mediate angiogenic sprouting. Moreover, infected or damaged tissues stimulate an adhesive, prothrombotic endothelial phenotype that orchestrates the vascular inflammatory response, including leukocyte recruitment and increased vascular permeability [3].

Cellular activation is often accompanied and partly driven by changes in cellular metabolism. Such tuning of metabolic responses provides energy and building blocks in the form of metabolic intermediates that fuel relevant biological processes [4, 5]. To this end, endothelial cell metabolism has been suggested as a potential target in conditions associated with pathological angiogenesis [6]. Whereas cytoplasmic 2‐phosphofructokinase‐6/fructose‐2,6‐bisphosphatase (PFKFB3)‐driven glycolysis facilitates sprouting angiogenesis, mitochondrial fatty acid oxidation (FAO) and glutamine metabolism are linked to proliferation in stalk cells and redox homeostasis in quiescent endothelial cells, respectively [7, 8, 9, 10].

Dysregulation of endothelial cell metabolism is also seen in several pathological conditions involving inflammation, including diabetes, atherosclerosis, and pulmonary arterial hypertension [11, 12, 13]. Stimulation with interleukin‐1β (IL‐1β) and tumor necrosis factor (TNF) increases PFKFB3‐driven glycolysis. However, the metabolic phenotype of inflammatory‐activated endothelial cells remains poorly defined. Here, we explored the apparent paradox that IL‐1β stimulation of human umbilical vein endothelial cells (HUVECs) inhibits proliferation, yet increases glycolysis. Characterizing the metabolic response of HUVECs over a period of 24 h, we observed that the increase in glycolysis was preceded by increased PFKFB3 transcription and translation. This enhanced glycolysis in response to IL‐1β stimulation was also associated with increased oxygen consumption rate (OCR), suggesting a general increase in cellular energy production, despite reduced cellular proliferation and a reduction in FAO. The IL‐1β‐induced metabolic response varied between donors, and this may be important if considering metabolic targeting endothelial cells in disease.

## Methods

### Reagents

Interleukin‐1β, EGF, bFGF, and VEGF 165 were from R&D systems (Minneapolis, MN, USA). Hydrocortisone, l‐glutamine, sodium pyruvate, glucose (45%), oligomycin, carbonyl cyanide‐4‐(trifluoromethoxy) phenylhydrazone (FCCP), and Dulbecco's Modified Eagle Medium (DMEM) without glucose (D5030) were from Sigma‐Aldrich (Milwaukee, WI, USA). FBS, gentamicin, fungizone, MCDB 131, and TRI reagent were from Thermo Fisher Scientific (Waltham, MA, USA). Trypsin and ethylenediaminetetraacetic acid tetrasodium salt dihydrate (EDTA) were from BioWhittaker (Walkersville, MD, USA). HEPES buffer was from Agilent (Santa Clara, CA, USA).

### Cell culture

Human umbilical vein endothelial cells were isolated as described [14] and cultured as previously described [15]. Briefly, cells were grown on polystyrene plastic coated with 0.1% gelatin, cultured in a 5% CO_2_ 95% humidity incubator at 37 °C, and used in passage 2–5. Cells were expanded in MCDB 131 medium supplemented with 7.5% FBS, 2 mm l‐glutamine, 10 ng·mL^−1^ rh‐EGF, 1 ng·mL^−1^ rh‐bFGF, 1 µg·mL^−1^ hydrocortisone, 50 µg·mL^−1^ gentamicin, and 250 ng·mL^−1^ fungizone. Prior to experiments, cells were cultured in MCDB131 as detailed, with some modifications and additions: 2% FBS, 5 ng·mL^−1^ rh‐EGF, 10 ng·mL^−1^ rh‐bFGF, 20 ng·mL^−1^ Long R3 IGF, 0.5 ng·mL^−1^ rh‐VEGF 165, and 1 µg·mL^−1^ ascorbic acid.

### Thymidine uptake, glycolysis, and fatty acid oxidation

Human umbilical vein endothelial cells were seeded at a density of 3.8 × 10^4^ cells/cm^2^ and cultured for 96 h to reach confluence. During the final 2 h of incubation, isotope‐labeled tracers (all from Perkin Elmer, Waltham, MA, USA), 5‐^3^H‐d‐glucose [0.4 µL·mL^−1^ (0.4 µCi·mL^−1^)], and ^3^H‐9‐10‐palmitic acid [0.4 µL·mL^−1^ (2 µCi·mL^−1^)] were added to the cultures to measure the rate of glycolysis and the rate of FAO, respectively, as described [9, 16]. When incubating with ^3^H‐9‐10‐palmitic acid, cells were co‐incubated with 50 µm carnitine (1 : 1000) and 100 µm cold palmitic acid. The rates of glycolysis and FAO were determined by measuring ^3^H transferred from the tracer to H_2_O by transferring supernatants to sealed glass vials. Evaporated H_2_O from supernatants was captured on Whatman paper in hanging wells over a 48 h of incubation at 37 °C to reach saturation, as described. The rate of glycolysis was calculated from the difference between the rate of ^3^H_2_O formation and the estimated rate of substrate recycling. The rate of ^3^H_2_O capturing had previously been calibrated using known amounts of ^3^H_2_O as a standard and according to [16]. ^3^H was detected by TriCarb 2810 TR Liquid Scintillation Analyzer (Perkin Elmer). Finally, to normalize for protein concentrations, cells were lysed in RIPA buffer and total protein concentration determined by bicinchoninic acid assay (BCA assay). Furthermore, cells were also incubated with ^3^H‐thymidine [1 µL·mL^−1^ (1 µCi·mL^−1^)] to determine proliferation rate as a result of DNA synthesis after lysing the cells in NaOH (0.2 m).

### TUNEL assay

Apoptosis was assessed by using TiterTACS In Situ Detection Kit (R&D Systems), a colorimetric‐based terminal deoxynucleotidyl transferase dUTP nick end labeling (TUNEL) assay following manufacturer's instructions. Cells were seeded into a gelatin‐coated 96‐well plate at a density of 3.8 × 10^4^ cells·cm^−2^ and cultured for 72 h before stimulation with IL‐1β for 24 h. Cells were also treated with staurosporine (0.5 µm) for 24 h to induce caspase 3 activation and induce apoptosis as described [17]. Briefly, cells were fixed in 4% buffered formaldehyde for 7 min, postfixed in 100% methanol for 20 min, and permeabilized by cytonin for 15 min. Cells were treated with TACS nuclease for 30 min at 37 °C to induce DNA fragmentation to validate assay. Cells were treated with 3% H_2_O_2_ in methanol for 5 min to quench endogenous peroxidase activity, next, incubated with labeling buffer for 5 min at room temperature, incubated with labeling reaction mix, or reaction mix without enzyme to measure background, for 1 h at 37 °C before adding Stop Buffer (5 min of RT). Plates were then incubated with Streptavidin‐HRP solution for 10 min at room temperature, incubated in the dark with TACS‐Sapphire for 30 min at room temperature, before stopping the reaction by adding 0.2 m HCl and measuring optical density (OD) at 450 nm using an Epoch microplate reader (BioTek, Winooski, VT, USA).

### Annexin V/propidium iodide assay

Cell viability was assessed using Annexin V/propidium iodide (PI) following manufacturer's protocol (BD Biosciences, Franklin Lakes, NJ, USA). HUVECs were seeded at 3.8 × 10^4^ cells·cm^−2^ in a gelatin‐coated 6‐cm cell culture dish and cultured for 72 h before 24 h of stimulation with IL‐1β. HUVECs treated with 500 mm H_2_O_2_ were used as a positive control. After treatment, cells were detached by 0.05% trypsin in EDTA and suspended in 10% FBS in PBS. Detached cells were pooled with the supernatants, washed once with 1× PBS, and finally resuspended in a 1× Annexin V binding buffer. Approximately 1 × 10^5^ cells/100 μL were transferred in 5‐mL flow cytometry tubes and stained with Annexin V‐FITC (5 μL per sample) and PI (5 μL per sample) at room temperature for 15 min in the dark. Postincubation, 400 μL of Annexin V binding buffer was added to each tube and samples were analyzed using FACSCantoII with facsdiva software (BD Biosciences). Apoptotic cells were defined as Annexin V^+^/PI^+^ population while live cells were defined as double‐negative cells.

### Glucose uptake

Human umbilical vein endothelial cells were seeded at 3.8 × 10^4^ cells·cm^−2^ in gelatin‐coated 24‐well plates, cultured for 96 h to reach confluence, and stimulated with IL‐1β (1 ng·mL^−1^) for 16 h. After 16 h, 0.5 µCi·mL^−1^ ^14^C‐2‐deoxy‐d‐glucose (Perkin Elmer) was added to the cells for 10 min at 37 °C, followed by washing three times in cold PBS and lysed in NaOH (0.2 m). Levels of ^14^C were measured by TriCarb 2810 TR Liquid Scintillation Analyzer (Perkin Elmer).

### BCA assay

The Pierce BCA assay microplate setup was used to measure protein concentration according to manufacturer's instructions (Thermo Fisher Scientific, https://assets.thermofisher.com/TFS‐Assets/LSG/manuals/MAN0011430_Pierce_BCA_Protein_Asy_UG.pdf). Briefly, BSA was diluted twofold from 2000 to 31 µg·mL^−1^ in PBS to generate a standard curve. Five microliters of standard or sample was added per well, mixed with 200 µL working reagent consisting of 50 parts solution A + 1 part solution B per well, and incubated for 20–30 min at 37 °C. Protein concentration was measured by OD at 562 nm.

### Protein gel separation and western blot

Protein gel separation and western blot were performed as previously described [15]. Cells were washed with cold PBS, followed by lysis using lysis buffer containing 10 mm Tris (pH 6.8), 5 mm EDTA, 6 mm NaF, 5 mm tetrasodium pyrophosphate (Na_4_P_2_O_7_), 2% SDS, as well as inhibitors of proteases (Sigma Aldrich, P5726, 1 : 100) and phosphatases (Sigma Aldrich, P8340, 1 : 100). Samples were prepared by adding sample buffer containing 72% glycerol, 28% β‐mercaptoethanol, 0.33 mg·mL^−1^ bromophenol blue at a 1 : 7 ratio (v/v), then heated to 65 °C for 10 min. 10 µg protein was loaded in each well in a 15‐well 10% or 4–20% polyacrylamide Bio‐Rad mini‐PROTEAN®TGX™ precast tris‐glycine gel; 5 µL of Amersham ECL rainbow marker–full range (Sigma Aldrich) was loaded to distinguish sizes. Separated proteins were transferred to a nitrocellulose membrane using the Trans‐blot® Turbo™ transfer system (Bio‐Rad) using the program “mixed MW.” Following transfer, membranes were first blocked with 5% no‐fat milk (Bio‐Rad, Hercules, CA, USA) in TBST (Tris‐buffered saline with 0.01% Tween 20, pH 7.4) for 30 min at RT. Next, membranes were incubated with primary antibodies overnight (4 °C), followed by washing with TBST and finally incubation with HRP‐conjugated secondary antibodies for 2 h. Substrate (SuperSignal™ West Dura Extended Duration Substrate; Thermo Fisher Scientific) was added to the membrane and product formation detected using ChemiDoc XRS+ system and Image Lab 4.1 (Bio‐Rad). When necessary, membranes were stripped using Restore PLUS western blot stripping buffer (Thermo Fisher Scientific) for 10 min then washed according to manufacturer's instructions, then blocked again, and stained with appropriate antibodies. Bands were quantified using volume tools in Image Lab 4.1 and normalized to β‐tubulin. All antibodies were diluted in 1% dry milk. Anti‐vascular cell adhesion molecule‐1 (VCAM‐1) (polyclonal BBA19, 1 : 1000) was purchased from R&D Systems; anti‐PFKFB3 (mAb clone EPR12594, 1 : 2000) and anti‐β‐tubulin (polyclonal ab6046, 1 : 20000) were purchased from Abcam (Cambridge, UK). HRP‐conjugated anti‐goat IgG (polyclonal sc‐2020, 1 : 10000) was purchased from Santa Cruz Biotechnology (Dallas, TX, USA). HRP‐conjugated anti‐rabbit IgG (polyclonal 711‐035‐153, 1 : 20000) was purchased from Jackson ImmunoResearch (West Grove, PA, USA).

### Reverse transcriptase quantitative PCR

RNA extraction and RT‐qPCR were performed as previously described [15]. Cells were washed in PBS at RT followed by lysis using Tri Reagent. RNA was extracted using 1‐bromo‐3‐chloropropane, isopropanol, and ethanol extraction, dissolving RNA in DEPC H_2_O. 1 µg cDNA was synthesized using SuperScript III® reverse transcriptase according to manufacturer's instructions then diluted 1 : 10 prior to PCR. Primers for *VCAM1* (5′–3′ AGTTGAAGGATGCGGGAGTAT, 5′–3′ GGATGCAAAATAGAGCACGAG 2.0 mm MgCl_2_), *PFKFB3* (5′–3′ GTCCCTTCTTTGCATCCTCTG, 5′–3′ CCTACCTGAAATGCCCTCTTC 1.5 mm MgCl_2_), and *HPRT* (5′–3′ AATACAAAGCCTAAGATGAGAGTTCAAGTTGAGTT, 5′–3′ CTATAGGCTCATAGTGCAAATAAACAGTTTAGGAAT, 2.0 mm MgCl_2_) were designed to span exon–exon junctions using Primer3 and checked for possible off‐target hybridization using BLAST. qPCR was performed using the AriaMX Real‐time PCR system (Agilent). Relative quantities were calculated using the ΔΔCT method, normalizing to HPRT.

### Metabolic assay

Cells were seeded 4.0 × 10^4^ cells per well in Seahorse XF24 polystyrene cell culture plates and cultured for 72 h before overnight stimulation with IL‐1β (1 ng·mL^−1^). The Seahorse cartridge was placed in calibrant solution overnight in a humidified non‐CO_2_ incubator at 37 °C overnight according to the manufacturer's instructions (https://www.agilent.com/en/products/cell‐analysis/how‐to‐run‐an‐assay). DMEM without NaHCO_3_ or phenol red supplemented with 2 mm l‐glutamine, 10 mm d‐glucose, 1 mm sodium pyruvate, and 5 mm HEPES buffer adjusted to pH 7.4 was used as Seahorse assay medium. For equilibration, cells were washed three times in Seahorse assay medium then incubated in humidified non‐CO_2_ incubator at 37 °C for 1 h. Metabolism was then analyzed using cell energy phenotype assay (Seahorse XFe24, Agilent). Basal extracellular acidification rate (ECAR) and OCR were measured in 5 cycles (2‐min mixing, 2‐min recovery, and 2‐min measurement) followed by simultaneous injection of oligomycin (2 mg·mL^−1^ well concentration) and FCCP (2 µm well concentration) followed by 5 measurement cycles. To allow cells to stabilize before measurements, only the last three measurements for each condition (basal and stressed, respectively) were included in the analysis. Medium was removed and cells were fixed in 0.5% periodate‐lysine‐paraformaldehyde (PLP) for 10 min, air‐dried, and then stored at 4 °C. Measurements were normalized to cell density by adding 120 µL 0.1% crystal violet in PBS to each well incubated at room temperature for 3–4 min followed by washing with tap water. Crystal violet was solubilized by adding 33% acetic acid, and OD was measured 550 nm using Epoch microplate reader (BioTek). Results were analyzed according to a protocol for cell energy phenotype (Agilent, https://www.agilent.com/cs/library/usermanuals/public/XF_Cell_Energy_Phenotype_Test_Kit_User_Guide.pdf).

### Lactate assay

Cells were seeded 3.8 × 10^4^ cells·cm^−2^ in gelatin‐coated 12‐well plates, cultured for 72 h to reach confluence. Cells were then stimulated with IL‐1β (1 ng·mL^−1^) for 20 h in experiment medium before changing to the Seahorse assay medium to avoid interference from phenol red and moved to a non‐CO_2_ incubator for 4 h. Supernatants were collected and frozen at −70 °C. Lactate concentrations were measured using Lactate Assay Kit II (Sigma Aldrich, MAK065) per manufacturer's instructions. Briefly, 25 µL sample was diluted 1 : 2 using lactate assay buffer in clear 96‐well plates; then, 50 µL of assay reagent was added per well. The plate was incubated in the dark at room temperature for 30 min. OD was measured using Epoch microplate reader (BioTek) at 450 nm. Concentration was calculated based on standard curve.

### Statistical methods

Statistical analyses were performed by applying graphpad prism (GraphPad, San Diego, CA, USA) version 8.1, using Student's *t* test unless otherwise noted. Wilcoxon signed ranked test was used where a normal distribution could not be assumed.

### Ethics statement

Human umbilical cords were collected after written informed consent by a protocol approved by the Regional Committee for Medical Research Ethics (2014/298 S‐05152a), Health Region South, Norway, conforming to the standards set by the Declaration of Helsinki.

## Results

### IL‐1β stimulation inhibits HUVEC proliferation rates

**Fig. 1:**
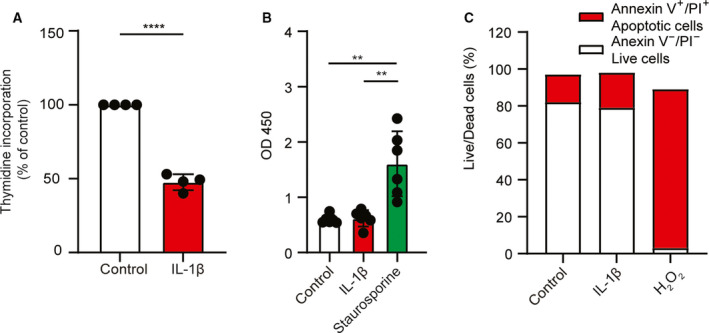
IL‐1β stimulation inhibits proliferation, but does not induce apoptosis. (A) Thymidine incorporation in IL‐1β‐stimulated HUVECs (1 ng·mL^−1^, 16 h; mean ± SD; *n* = 4 donors). (B) Colorimetric TUNEL assay in IL‐1β‐stimulated HUVECs (1 ng·mL^−1^, 24 h), with staurosporine (0.5 µm) as positive control (mean ± SD; *n* = 6 donors). (C) Flow cytometry of Annexin V‐FITC/PI‐stained IL‐1β‐stimulated HUVECs (1 ng·mL^−1^, 24 h), with H_2_O_2_ (500 mm) as positive control, showing the percentage of live (Annexin V^−^ PI^−^) and apoptotic or dead (Annexin V^+^ PI^+^) cells (*n* = 1 donor). **P < 0.01, ****P < 0.0001.

We have previously observed that IL‐1β stimulation inhibits proliferation of cultured human endothelial cells, as measured by an increase in cell numbers over time [18]. Cellular proliferation rates often correlate closely with cellular glycolytic activity [19], as cell division requires energy and metabolic building blocks. Nevertheless, in addition to inhibiting cell proliferation, IL‐1β stimulation also increases glycolysis in confluent endothelial cultures [20]. To explore this apparent paradox, we first confirmed the effect of IL‐1β on proliferation in confluent HUVECs by measuring thymidine incorporation 16 h after IL‐1β stimulation. In line with previous findings, we observed that IL‐1β reduced proliferation in HUVECs by approximately 50% (Fig. 1A). For this experiment, HUVECs were cultured for 96 h after seeding, reflecting the conditions under which IL‐1β stimulates endothelial glycolysis [20]. At this stage, HUVECs were confluent, but not fully contact inhibited, as demonstrated by the rate of thymidine incorporation in unstimulated cells (Fig. 1A). We have previously demonstrated that IL‐1β does not induce cell death in HUVECs, as measured by release of lactate dehydrogenase [15]. To strengthen the evidence that the reduced cell proliferation was not due to cytotoxic effects, we also measured apoptosis by assessing DNA fragmentation in a TUNEL assay. We found no difference between control and IL‐1β‐stimulated cells, while staurosporine significantly increased apoptosis as reported (Fig. 1B) [17]. Moreover, Annexin V/PI staining showed no difference in the level of early apoptotic events between IL‐1β‐stimulated and control cells (Fig. 1C).

### IL‐1β stimulation increases HUVEC glucose uptake, glycolysis, and PFKFB3 expression

**Fig. 2:**
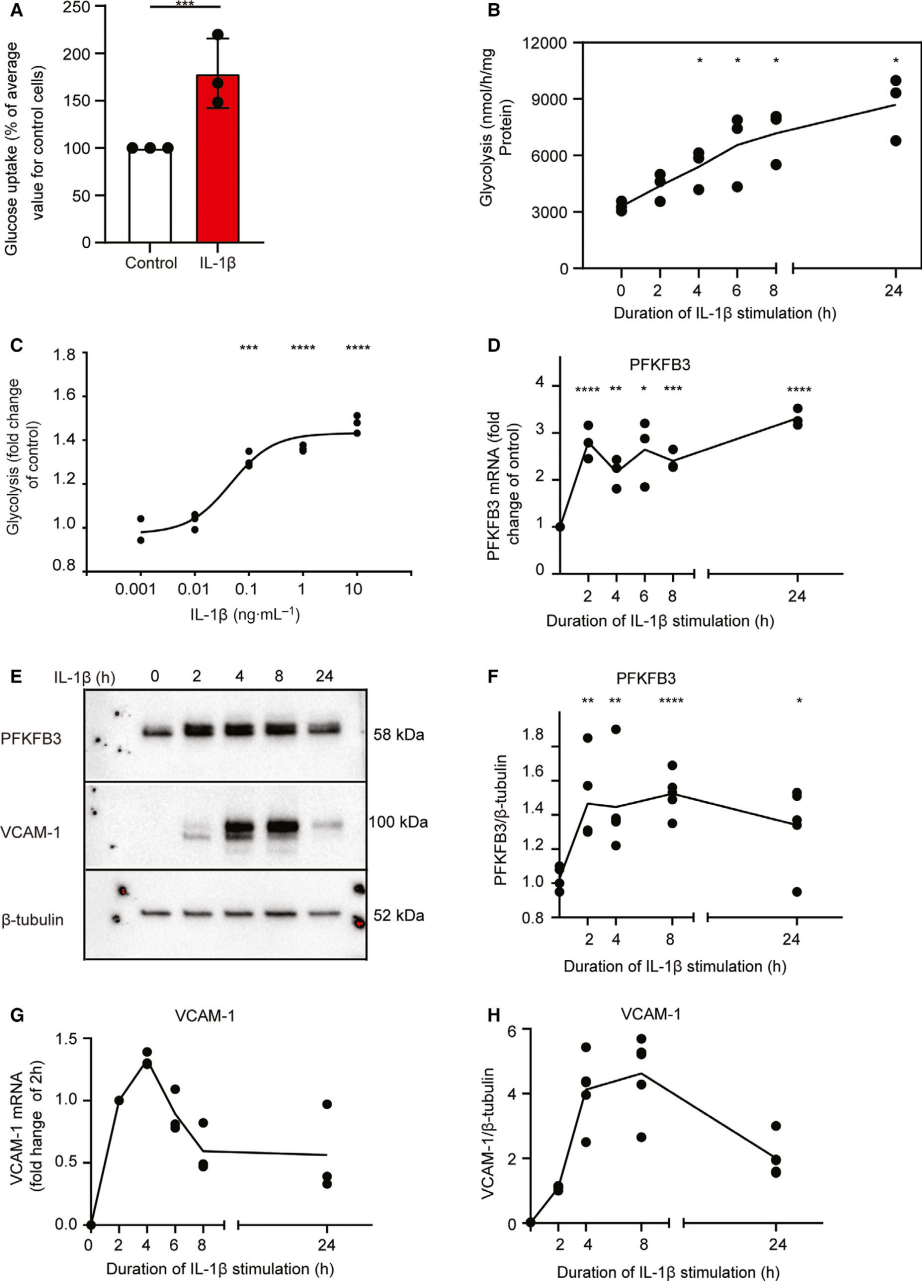
IL‐1β stimulation increases endothelial glucose uptake, glycolysis, and PFKFB3 expression. (A) Uptake of ^14^C‐2‐deoxy‐d‐glucose in IL‐1β‐stimulated HUVECs (1 ng·mL^−1^, 16 h; mean ± SD; *n* = 3 donors). (B) Rate of glycolysis in IL‐1β‐stimulated HUVECs (*n* = 3 donors). (C) Glycolysis fold change 6 h after stimulation with different doses of IL‐1β (*n* = 3 donors). (D) RT‐qPCR of *PFKFB3* following in IL‐1β‐stimulated HUVECs (1 ng·mL^−1^) (*n* = 3 donors). (E) Representative immunoblot for PFKFB3 (top), VCAM‐1 (middle) and β‐tubulin loading control (bottom) in IL‐1β‐stimulated HUVECs (1 ng·mL^−1^, 2–24 h). (F) Densitometric quantification of PFKFB3 relative to β‐tubulin loading control (*n* = 5 donors). (G) RT‐qPCR of VCAM‐1 in IL‐1β‐stimulated HUVECs (1 ng·mL^−1^, 2–24 h) (*n* = 3 donors). (H) Densitometric quantification of VCAM‐1 relative to β‐tubulin loading control (*n* = 5 donors). B–D and F–H: lines show means, and dots show individual donors. *P < 0.05, **P < 0.01, ***P < 0.001, ****P < 0.0001.

Supporting the previously reported augmentation of endothelial cell glycolysis by inflammatory cytokines in our system [20], we next measured glucose uptake and found an almost twofold increase in IL‐1β‐stimulated cells compared with control (Fig. 2A). Expanding on previous studies, we also explored the kinetics of the glycolytic response and found that IL‐1β stimulation gradually increased glycolysis over the course of 24 h (Fig. 2B). The response was dose‐dependent and peaked at 1 ng·mL^−1^ (Fig. 1C), similar to the upregulation of adhesion molecules [18]. Next, we attempted to map the regulation of the glycolytic machinery by IL‐1β over time, focusing on expression of the glycolytic enzyme PFKFB3. PFKFB3 is responsible for the synthesis of fructose 2,6‐bisphosphate (F‐2,6‐BP), the major allosteric activator of phosphofructokinase 1 (PFK‐1), a key rate‐limiting enzyme of glycolysis [21]. PFKFB3 expression correlates with glycolytic activity in endothelial cells [16, 22, 23], and its transcription increases following IL‐1β stimulation [20]. In line with this, we found that *PFKFB3* mRNA expression rapidly increased within 2 h of IL‐1β stimulation (Fig. 2D). Adding to previous data, we also found that transcription levels peaked at this time point and remained elevated until 24 h (Fig. 2D). Moreover, we measured PFFKB3 protein and observed kinetics similar to the transcriptional response (Fig. 2E, upper panel, densitometry shown in Fig. 2F). VCAM‐1 expression served as positive control and followed the expected pattern with an acute increase in expression within 2 h, a peak at 4–6 h, and a decline within 24 h after IL‐1β stimulation (Fig. 2E, middle panel, Fig. 2G,H) [24].

### IL‐1β stimulation is associated with increased ECAR without affecting extracellular lactate accumulation and increases OCR in the presence of reduced fatty acid oxidation

**Fig. 3:**
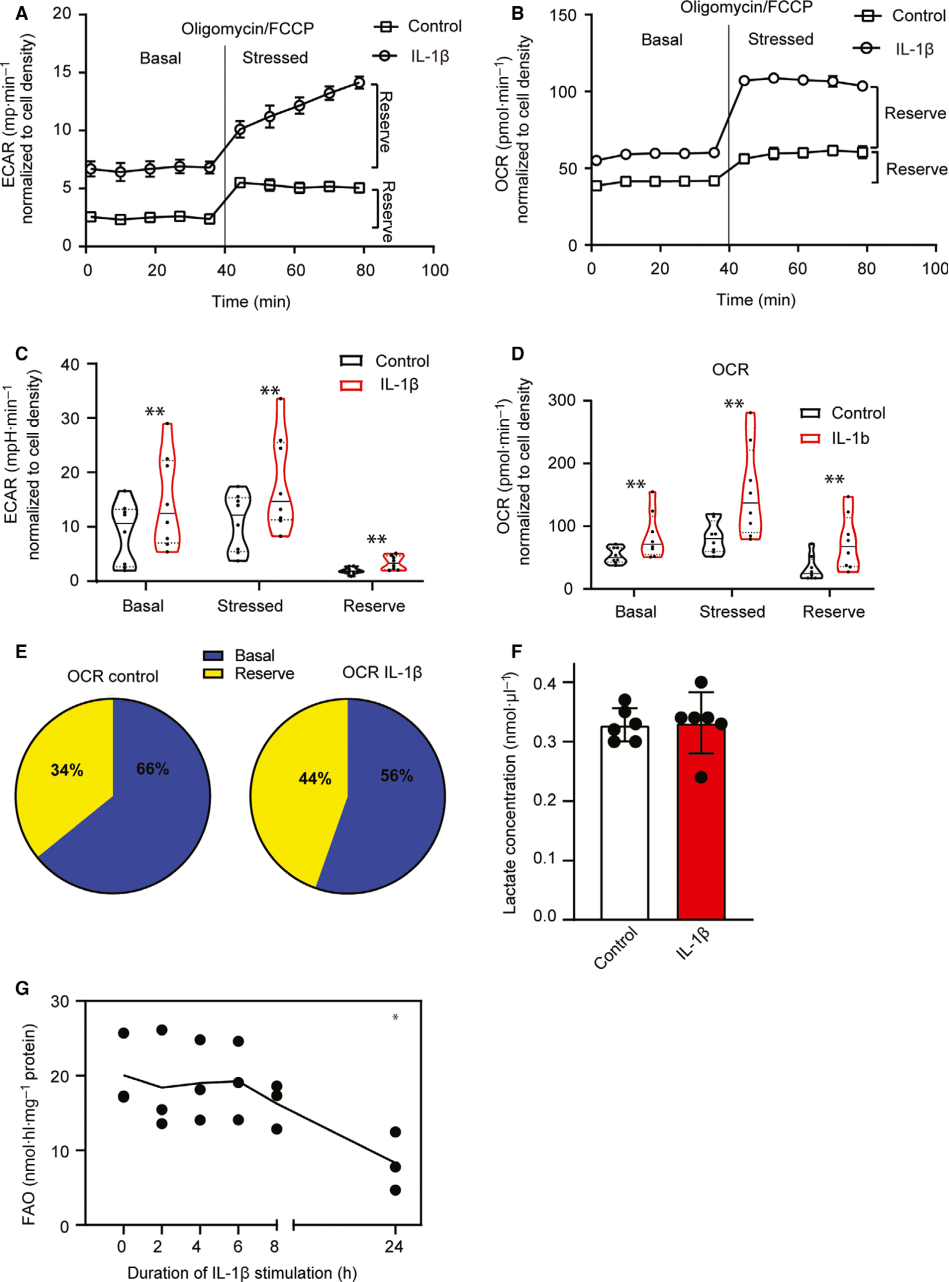
IL‐1β‐dependent glycolysis correlates with increased ECAR and oxygen consumption, but not with increased lactate production. HUVECs were stimulated with IL‐1β (1 ng·mL^−1^, 16 h) and analyzed in the Seahorse XF system. (A, B) Graphs from one representative Seahorse experiment showing ECAR (A) and OCR (B) in unstimulated (square) and IL‐1β‐stimulated HUVECs (circle) normalized to cell density. (C, D) Violin plots showing the distribution of basal, stressed, and reserve capacity ECAR (C) and OCR (D) in unstimulated and IL‐1β‐stimulated HUVECs (25% and 75% quartiles: dotted lines, median: solid line) (*n* = 8 donors). **P = 0.0078 Wilcoxon matched‐pairs signed‐rank test. (E) Pie chart showing ratio of basal and reserve OCR in unstimulated (95% CI; basal, 65–68, reserve, 3–5) and IL‐1β‐stimulated HUVECs (95% CI: basal, 55–58, reserve 42–45). (F) Extracellular lactate concentration in IL‐1β‐stimulated HUVECs (mean ± SD; *n* = 6 donors). (G) FAO in IL‐1β‐stimulated HUVECs (mean of 3 donors and dots marking independent donors). *P < 0.05.

To better understand the metabolic adaptations of IL‐1β‐stimulated HUVECs, we next measured ECAR and OCR using the cell energy phenotype test. Briefly, to induce a stressed metabolic phenotype that reveals the maximal metabolic potential of the cells, ECAR and OCR were measured before (basal) and after (stressed) simultaneous injection of the ATP synthetase inhibitor oligomycin and the mitochondrial uncoupler FCCP. Figure 3A,B shows ECAR and OCR in one representative Seahorse experiment, demonstrating an increase in metabolic activity and maximal potential following IL‐1β stimulation. Figure 3C,D plots the mean and quartile ranges of basal, stressed, and reserve capacity (the difference between stressed and basal) ECAR and OCR, respectively, in control and IL‐1β‐stimulated HUVECs from eight donors. In line with our findings of increased glycolytic flux, we observed an increase in both basal and stressed ECAR in IL‐1β‐stimulated HUVECs (Fig. 3A,C). However, we also observed a rise in both basal and stressed OCR (Fig. 3B,D), suggesting that the increased glycolytic flux in IL‐1β‐stimulated HUVECs did not represent a shift from oxidative to glycolytic metabolism but rather a general increase in metabolic activity. Moreover, IL‐1β increased the OCR reserve capacity, that is, the percentage of total oxygen consumptive capacity that is kept unused and can be mobilized in response to increased metabolic demands (Fig. 3E). We next measured extracellular lactate concentration and found that IL‐1β‐stimulated HUVECs, despite increased glycolytic rate and ECAR, did not produce more extracellular lactate than control HUVECs (Fig. 3F). Increased mitochondrial activity may be associated with FAO [9, 25]. However, after IL‐1β stimulation, FAO remained stable for the first 8 h and decreased between 8 and 24 h (Fig. 3G). Taken together, we show that IL‐1β stimulation of HUVECs increased both ECAR and OCR without increasing extracellular lactate levels, suggesting that pyruvate produced by increased glycolytic activity is shunted into the tricarboxylic acid (TCA) cycle to support increased oxygen consumption in IL‐1β‐stimulated HUVECs.

### The increase in ECAR and OCR in response to IL‐1β varies between donors

**Fig. 4:**
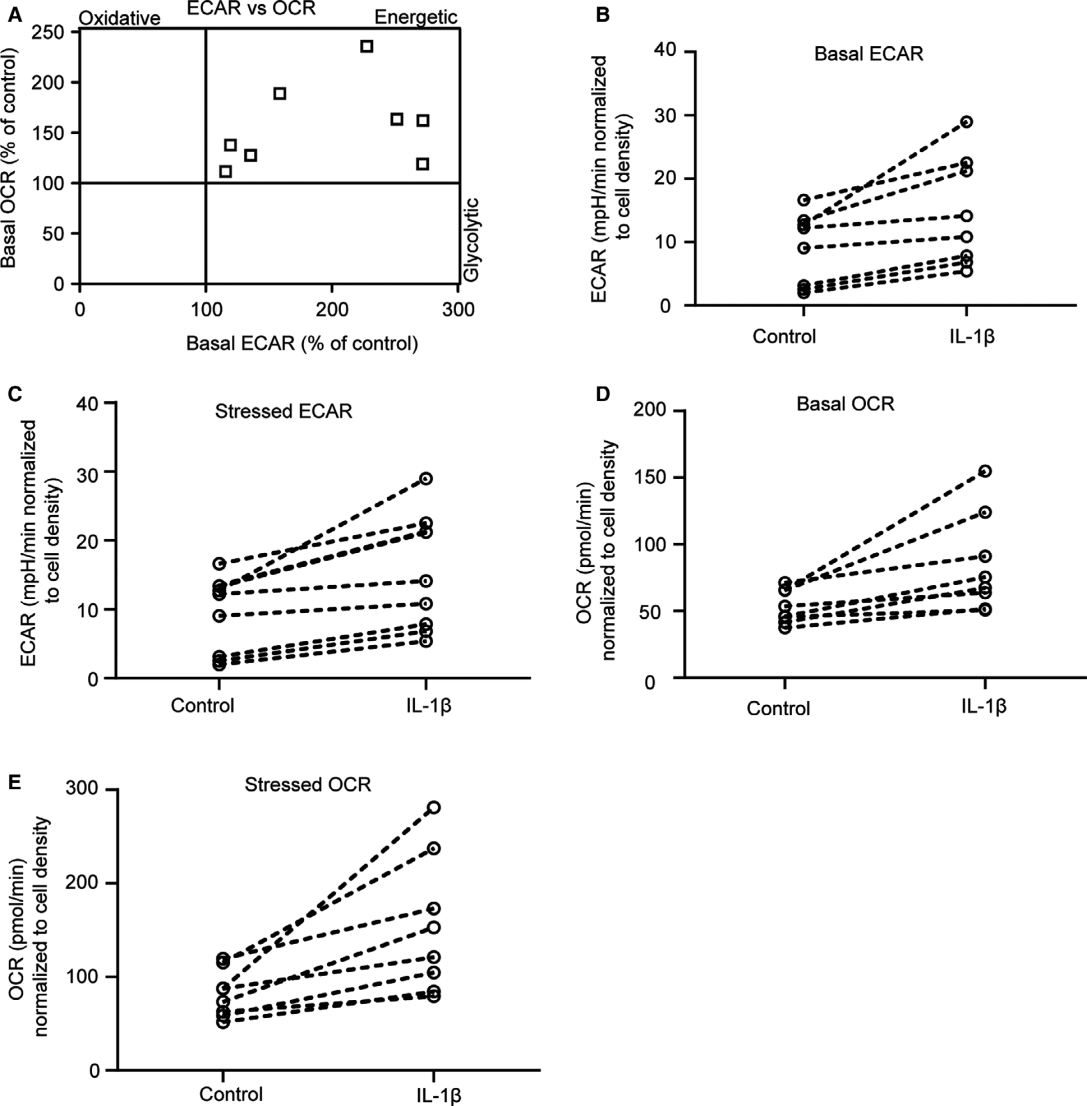
The increase in ECAR and OCR in response to IL‐1β varies between donors. This figure shows the same data as Fig. 3C,D, but illustrates the relationship between results from individual donors (*n* = 8). (A) Relationship between basal ECAR and basal OCR in IL‐1β‐stimulated HUVECs relative to control (defined as 100%). Basal (B) and stressed (C) ECAR in IL‐1β‐stimulated HUVECs, dotted lines connect samples from the same donor. Basal (D) and stressed (E) OCR in IL‐1β‐stimulated HUVECs, dotted lines connect samples from the same donor.

Despite the fact that lactate production was unaffected by IL‐1β stimulation, ECAR was increased, suggesting CO_2_‐dependent acidification [26]. In line with this hypothesis, the magnitude of the IL‐1β‐stimulated increase in ECAR correlated with the increase in OCR in HUVECs from most of the eight donors used for the experiment (4A). However, the cells from at least one donor diverged from this general pattern and showed a prominent rise in ECAR without concomitant increase in OCR (Fig. 4A). Cells from this donor were not available to measure lactate concentrations. We also observed a marked variation in the amplitude of response to IL‐1β stimulation for both basal (Fig. 4B) and stressed (Fig. 4C) ECAR, and basal (Fig. 4D) and stressed (Fig. 4E) OCR.

## Discussion

In this study, we show that inflammatory activation of endothelial cells leads to a generalized change in cellular metabolism, shifting toward a phenotype with increased cytoplasmic glycolysis and oxygen consumption. The increased glycolysis correlated with a rapid and sustained upregulation of the glycolytic enzyme PFKFB3, suggesting mobilization of the glycolytic machinery. However, extracellular lactate concentrations remained stable, suggesting that pyruvate was shunted into the TCA cycle to provide fuel for oxidative metabolism and, possibly, the generation of metabolic intermediates [27].

The shift toward this metabolically active phenotype happened despite a 50% reduction in DNA synthesis, indicating reduced cellular proliferation. We also observed a corresponding reduction in FAO, which supports DNA synthesis as well as redox balance in endothelial cells [9, 10]. The combination of reduced proliferation and high metabolic activity is of interest, because proliferation is an energy‐demanding process that often drives catabolic metabolism. Nevertheless, our results suggest that other biological processes in endothelial cells may create a need for the switch to the more metabolically active phenotype that occurs upon inflammatory activation. To some extent, this is similar to the situation in angiogenic endothelial cells where nonproliferating, migratory tip cells rely on increased PFKFB3‐driven glycolysis localizing to sites for F‐actin polymerization, while proliferating stalk cells rely on glutamine for producing biomass through the TCA cycle and finally fatty acids for dNTP synthesis [7, 8, 9].

The contribution of PFKFB3 to endothelial cell glycolysis has been well‐documented in angiogenesis [16, 22, 23]. It is therefore not surprising that PFKFB3 is also upregulated as part of the glycolytic response to IL‐1β [20]. Adding to previous reports, we found that protein levels of PFKFB3 reached their peak 2 h after IL‐1β stimulation, in other words before the rate of glycolysis was significantly enhanced. This apparent lag between the increase in PFKFB3 and the boost in glycolysis could be explained by the time it takes for the PFKFB3 product F‐2,6‐BP to accumulate sufficiently to boost PFK‐1 activity [28]. However, additional interplay with other regulatory mechanisms cannot be excluded.

We also observed that the increase in glycolysis did not correlate with increased lactate production, but rather with an increase in both basal OCR and OCR reserve capacity. This increase in OCR was unexpected and challenges the assumption that the TCA cycle minimally contributes to energy homeostasis in endothelial cells [8]. Our findings are also different from the situation in macrophages, where inflammatory activation by endotoxin increases glycolysis, but reduces oxygen consumption [29]. We observed a reduced rate of FAO, based on measuring ^3^H 9‐10‐palmitic acid flux, suggesting that substrates other than fatty acids are used to fuel the TCA cycle and oxidative phosphorylation in IL‐1β‐stimulated cells. Indeed, this agrees with previous studies that have identified key roles for fatty acids in endothelial cell generation of dNTP and NADPH, supporting cellular proliferation and redox homeostasis, rather than fueling oxidative phosphorylation and energy production [9, 10]. While we did not explore the fate of glutamine, our results suggest that at least part of the increase in oxidative metabolism relies on the increased availability of glucose‐derived pyruvate resulting from IL‐1β‐stimulated glycolysis.

The entry of pyruvate into the TCA cycle allows its oxidation, thus generating more energy than if pyruvate is converted to lactate and secreted. In fact, the nonoxygen‐dependent form of glycolysis only yields two molecules of ATP per glucose molecule, whereas complete combustion of glucose may yield up to 36 ATP molecules per glucose molecule [30]. In future studies, it would be interesting to address the contribution of glucose‐derived pyruvate in the TCA cycle further, to determine whether this increased flux may also contribute to the production of metabolic intermediates that could regulate endothelial activation, as shown for glutamine‐derived succinate in macrophages [29].

In most donors, the observed increase in ECAR correlated with the increase in OCR, rather than with extracellular lactate concentrations. Despite a common association of ECAR to glycolysis and lactate secretion, it may also be driven by CO_2_ production from cellular respiration [26]. For example, in mouse podocytes, blocking glycolysis with 2‐deoxy‐d‐glucose or lactate production with oxamate increases ECAR by increasing CO_2_ production [31]. Nevertheless, one donor stood out, by showing a minimal increase in OCR, but a large increase in ECAR, suggestive of a predominantly glycolytic response. There was also marked donor variation in the amplitude of response to IL‐1β stimulation. While cells from all donors showed a metabolic shift toward higher metabolic activity, the shift was very modest in some. It is well known that primary human endothelial cells from different donors show heterogeneous responses [32]. We suggest that these donor differences may be important when considering metabolism as therapeutic targets.

An important question not addressed by our current study is whether the endothelial metabolic response to inflammatory stimulation is similar in endothelial cell subsets from other vascular beds. Endothelial cells lining arteries, capillaries, and veins are exposed to very different oxygen partial pressures and are also exposed to different microenvironmental conditions in different parts of the body [33]. For example, human dermal microvascular endothelial cells undergo metabolic reprogramming under hypoxic conditions [34], but it is not known how the metabolism of these cells change compared with HUVECs or in response to cytokine stimulation. Future studies should aim to understand the molecular basis for the donor variance in metabolic responses and also characterize responses between different endothelial subsets from different vascular beds.

## Author contribution

JAW, BS, GH, and JHF conceived and designed the study; JAW, DP, SK, and JHF collected the data; JAW, BS, SK, and JHF performed the analyses; JAW wrote the manuscript under supervision of JHF, BSS and GH, and DP and SK took part in amending and proofreading the final manuscript.

## Conflict of interest

The authors declare no conflict of interest.

## Data Availability

The authors confirm that the data supporting the findings of this study are available within the article.
